# Enhancing rapid nitrogen freezer performance for Litchi fruit: a Taguchi-based approach to reduce cracking, time, and nitrogen consumption

**DOI:** 10.1038/s41598-025-95215-3

**Published:** 2025-04-01

**Authors:** Mohamed Anwer Abdeen, Zeyong Zheng, Xiaojin Cheng, Baiqing Yu, Chongyang Han, Yong Cao, Wenquan Huang, Ali Salem, Alaa Awny, Weibin Wu

**Affiliations:** 1 College of Engineering, South China Agricultural University, Guangzhou, 510642 China; 2 Agricultural Engineering Department, College of Agriculture, Zagazig University, Zagazig, 44519 Egypt; 3 Guangdong Provincial Key Laboratory of Nutraceuticals and Functional Foods, College of Food Science, South China Agricultural University, Guangzhou, China; 4 Civil Engineering Department, Faculty of Engineering, Minia University, Minia 61111, Egypt; 5 Structural Diagnostics and Analysis Research Group, Faculty of Engineering and Information Technology, University of Pécs, Pécs 7622, Hungary

**Keywords:** Nitrogen quick freezing, Litchi fruit, Food processing, Freezing tunnel, Fruit crack, Taguchi method, Environmental impact, Energy science and technology, Mechanical engineering, Engineering, Software

## Abstract

This study employed the Taguchi technique to investigate the influence of various liquid nitrogen quick-freezing parameters on litchi fruit cracking, nitrogen consumption, and freezing time. The experiments included testing different freezing temperatures (− 40, − 50, − 60, and − 70 °C), two types of nitrogen spraying nozzles (Hollow-cone and Full-cone), and two fan speeds (800 and 1200 rpm). Before freezing, the litchis were soaked in a solution and precooled as a pre-treatment to mitigate peel cracking and preserve quality. The results revealed that the crack ratio, freezing time, and nitrogen consumption decreased as temperature and fan speed increased. Among the nozzle types, Full-cone nozzles exhibited superior performance, achieving reductions of more than 15% in crack ratio, over 8% in freezing time, and more than 4% in nitrogen consumption compared to Hollow-cone nozzles. The predicted values derived from the Taguchi method showed strong alignment with the experimental data, validating the robustness of the optimization approach. This study contributes novel insights to the field of food freezing technology by introducing an innovative method for minimizing fruit cracking during freezing without the need for packaging. The findings also highlight the potential for reducing freezing time, operational costs, and nitrogen usage, offering practical implications for the food processing industry.

## Introduction

Fruits are essential in the human diet as they are rich in nutrients, calories, and natural sugar. They also support heart and brain health, enhance immunity, and prevent cancers^1^. Fruits are rich in fibers that regularize digestion, manage weight and cholesterol values, and control blood sugar^2^. Litchi (*chinensis Sonn*.) is a sub-tropical fruit of high commercial value, which is cherished for its unique flavor, texture, and color^3,4^.

China is the largest Litchi producer, accounting for over 70% of global production, and has great potential as a Litchi exporter. However, Litchi fruit is a highly perishable commodity with less than 30 days shelf-life using modern preservation technologies, and thus, only a small proportion of fresh Litchis are processed^5^. The lack of availability of a continuous cold chain, damage during transportation, and poor local storage infrastructure further aggravate these issues, and postharvest Litchi losses are estimated to be 25 to 30% or even higher in some growing areas^6^.

Preserving food to extend its shelf-life while guaranteeing its quality is a fundamental concern in the food industry^7^. Freezing fruits before storing them is useful in saving fruits for a long time^8–10^. Freezing is the foundation and core technology of food preservation. Freezing extends the products’ shelf life by decreasing water activity, enzyme reactions, and microbial activity^11,12^. The low temperature during freezing helps preserve the bioactive compounds and nutrients^13^. Recently, there has been an increasing demand for frozen vegetables and fruits^14^. Frozen storage is a useful alternative for the long-term preservation of fruits^9,10,15^. The freezing rate is the most important factor influencing the quality of frozen food^16^. When the freezing rate is slow, large ice crystals form. These large ice crystals can cause substantial damage to the food’s cell structure, leading to a loss in texture and quality during the thawing process^17^. On the other hand, when the freezing rate is fast, small ice crystals are formed. These small ice crystals are distributed more homogeneously and evenly within the food structure, causing minimal damage to the tissue. This improves food texture and quality preservation during thawing.

Liquid nitrogen spray freezing (NF) takes advantage of the enormous heat absorbed during gasification. With rapid heat transfer or fast freezing rate, the time of a food product within the maximal crystal forming temperature range during the NF process is short, resulting in small and uniformly distributed ice crystals^18,19^. Sanchez-Alonso et al.^20^ observed that NF showed better results in cod quick freezing than in blast freezing and contact small space freezing. Lopkulkiaert et al.^21^ demonstrated that NF is significantly superior to air-cooling and shelf-contact freezing in terms of freezing rate and moisture retention in a quick-freezing study of *Penaeus vannamei*.

To preserve the original color of litchi fruits, some researchers have developed pre-treatment protocols prior to freezing. Liang et al.^22^ implemented a comprehensive pre-cooling and treatment process. They initially precooled litchi fruits to 10 °C using an ice-water mixture, transported them to the laboratory, and stored them at 4 °C for 6 h before applying color-preserving treatments. After precooling, the fruits were dipped in boiled water for 7 s, followed by dipping in cold water for 1 min and then immersed in a solution containing 50 g/L citric acid and 20 g/L sodium chloride for 2 min. Subsequently, 100 g batches of treated fruits were vacuum-bagged (−0.09 MPa) in nylon composite polyethylene bags (one sample per bag) and pre-cooled in a refrigerator until the internal temperature reached 0 °C (± 1 °C) before freezing. Similarly, Ducamp-Collin et al.^23^ focused on maintaining the red pericarp color of harvested litchis. They cooled and transported fresh litchis in 10 °C cool boxes to the experimental site. Upon arrival, the fruits were immersed in a solution containing 7.5 g/L chitosan and 600 g/L citric acid at room temperature (20 °C), dried, and stored at 4 °C until the freezing experiments commenced.

The disadvantages of liquid nitrogen spraying to freeze food are: (1) the freezing cost is high due to the loss of liquid nitrogen consumption and cold capacity, and (2) the freezing speed is extremely fast; There will be a large instantaneous temperature difference between the surface and the center of the food, and the expansion pressure is large, resulting in food cracking, so it is not suitable to freeze foods that are too thick and too large, and the thickness should be less than 10 cm^24^. In order to solve the problem of low-temperature fracture of food caused by the low temperature of liquid nitrogen, Baolin Liu et al.^25^ designed a new fluidized bed quick-freezing device that can control the air temperature in the bed by controlling the evaporation of liquid nitrogen spray for different foods, which can realize the partial vitrification and freezing of food to a certain extent and will not cause low-temperature fracture of food due to low temperature, but further research is needed to solve these three shortcomings completely.

Dipping fruits in dry sugar or syrups is a traditional pre-treatment to preserve color, flavor, texture, and vitamin C content and prevent freezing-thawing fruits’ browning. Syrup concentrations between 20% and 65% are generally employed, although 40% syrup is enough for most fruits. Sucrose is the osmotic agent most suitable for fruits, although other substances, including sucrose, Glucose, fructose, lactose, l-lysine, glycerol, polyols, maltodextrin, starch syrup, or combinations of these solutes, can be used^26,27^. Osmotic dehydration is the phenomenon of water removal from lower concentration to higher concentration solute through a permeable membrane, resulting in the equilibrium condition on both sides of the membrane^28^. It has been found to have wide applications in preserving food materials since it lowers the water activity of fruits and vegetables. Osmotic dehydration is preferred over other methods due to its color, aroma, nutritional constituents, and flavor compound retention value^29^.

Based on previous reviews, it is evident that litchis play a significant role in China’s national income and industry due to their high production volume and global export market. However, its commercial potential is hindered by its short shelf life and the rapid deterioration of its fresh pericarp color. Additionally, the conventional freezing process often results in excessive nitrogen consumption and frequent cracking of the fruit peel, further limiting its marketability. To address these challenges, this study investigated the effects of freezing temperatures, pre-treatment methods, fan speeds, and spraying nozzles type on optimizing the freezing process. The primary objectives were to minimize the crack ratio, reduce freezing time, and lower nitrogen consumption, thereby enhancing the efficiency and quality of frozen litchi production.

## Materials and methods

### Freezing tunnel

**Fig. 1:**
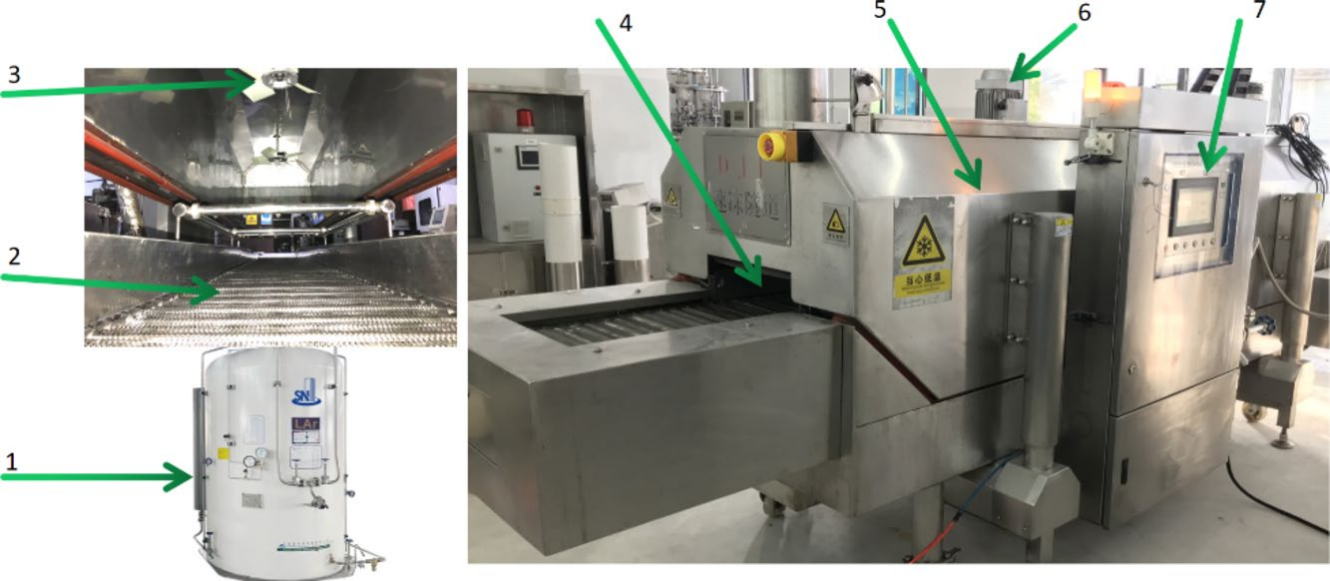
Freezing tunnel. (1) nitrogen tank; (2) metal mesh feeding chain; (3) distributing fan; (4) freezing tunnel; (5) tunnel cover; (6) electric motor; (7) plc unit.

**Fig. 2:**
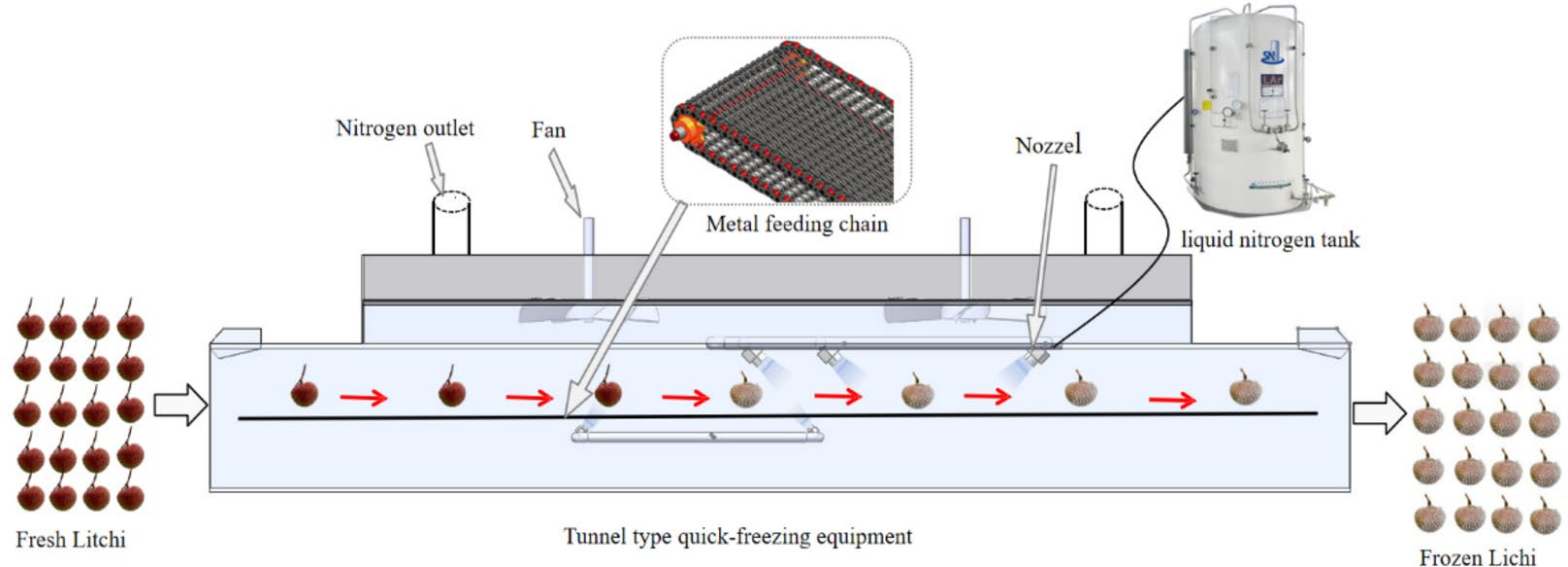
Workflow diagram of the freezing process.

The experiments utilized a liquid nitrogen freezing unit manufactured by Shenzhen De jie li Cryogenic Technology Co., Ltd. Formula. It comprises a feeding chain, freezing tunnel, PLC control unit, tunnel cover, distributing fans, nitrogen tank, frame, transmission system, and electric motors (Fig. 1). The workflow diagram of the freezing process is shown in Fig. 2.

### Lithi core temperature monitoring system

**Fig. 3:**
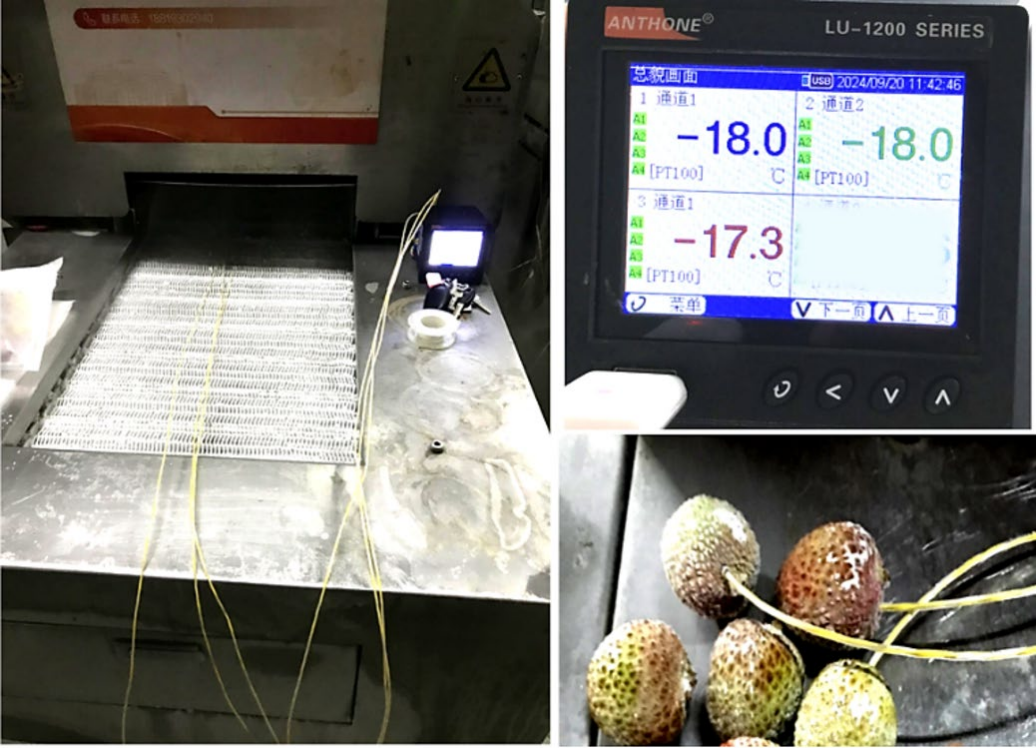
Fruit core temperature monitoring.

A core temperature monitoring system was used to measure the core temperature change of Litchi fruits during the freezing process (Fig. 3). The system consisted of 3 dip temperature sensors (PT100 type, 5000 mm wire length, temperature range of − 200: 50 ºC) connected to a 4 channels data logger (Anthone lu-1200 series). The temperature change data were recorded on a USB disk as an Excel sheet. The Litchis were frozen until their core temperature reached − 18 ºC.

### Litchi fruit preparation and pre-treatments

The ripened Litchi fruits (*Feizixiao*) were obtained from an orchard in Chaozhou, China, and selected according to the similarity and uniformity of shapes, colors, and sizes. The average fruit weight was 23.9 g, the average height was 36.09 mm, and the average diameter was 35.2 mm.

The selected fruits were dipped in the prepared solution of 50 g/L citric acid, 20 g/L sodium chloride^23^, and 50 g/l Glucose^26,27,29^ for different periods (2, 15, 30, and 60 min) and precooled in the refrigerator until their core temperature reached 4 ± 1 and 0 ± 1 ºC as pre-experimental treatments. The litchi core temperature was monitored by the core temperature monitoring system illustrated in Fig. 3. Subsequently, a series of freezing experiments were conducted to establish the optimal experimental freezing temperature range and evaluate the impact of pre-treatment methods on the crack ratio of litchi fruit peel. Same pre-experiments were conducted using fresh fruits without subjecting them to the dipping pretreatment. This was done to isolate and evaluate the specific impact of the dipping pretreatment on the results.

The most effective pre-treatments (see Table 1) were achieved by dipping the fruits in the solution for 30 min and pre-cooling them until the core temperature reached 0 ± 1 °C. Additionally, the highest temperature could be tested is −70 °C, as −80 °C caused over 60% cracking in the fruit peel, making it unsuitable for further experimentation.

**Table 1:** Pretreatment data.

Freezing temperature, °C	2 min dipping	15 min dipping	30 min dipping	60 min dipping	Un dipped fruits
− 40	13.33	6.67	0	3.33	13.33
− 50	15	13.33	6.67	8	15
− 60	20	20	13.33	20	26.67
− 70	35	26.67	20	26.67	35

### Taguchi’s experiment design

The Taguchi method is an orthogonal statistical method developed by Genichi Taguchi to improve production quality. It is being applied in engineering analysis^30^and biotechnology^31,32^to analyze the parameters’ effect on the experimental results. This method reduces test number and the uncontrolled factors that affect the experiment, resulting in time and cost savings^33,34^. It utilizes a loss function in measuring the deviation between the experimental and desired values, which is then converted into a signal-to-noise ratio (S/N)^34,35^. The S/N is divided into three categories, as shown by the following Eqs. (1–3):

The nominal is the best:

1 $$\:\frac{S}{N}=10\:\text{l}\text{o}\text{g}\frac{\bar{\text{y}}}{{s}_{y}^{2}}.$$

The higher, the better.

2 $$\:\frac{S}{N}=-\text{l}\text{o}\text{g}\frac{1}{n}\left(\sum\:\frac{1}{{y}^{2}}\right).$$

The lower, the better.

3 $$\:\frac{S}{N}=-10\:\text{l}\text{o}\text{g}\frac{1}{n}\left(\sum\:{y}^{2}\right),$$

where ӯ: observed data average; $\:{s}_{y}^{2}$: y variation; n: observations number; y: observed data.

**Fig. 4:**
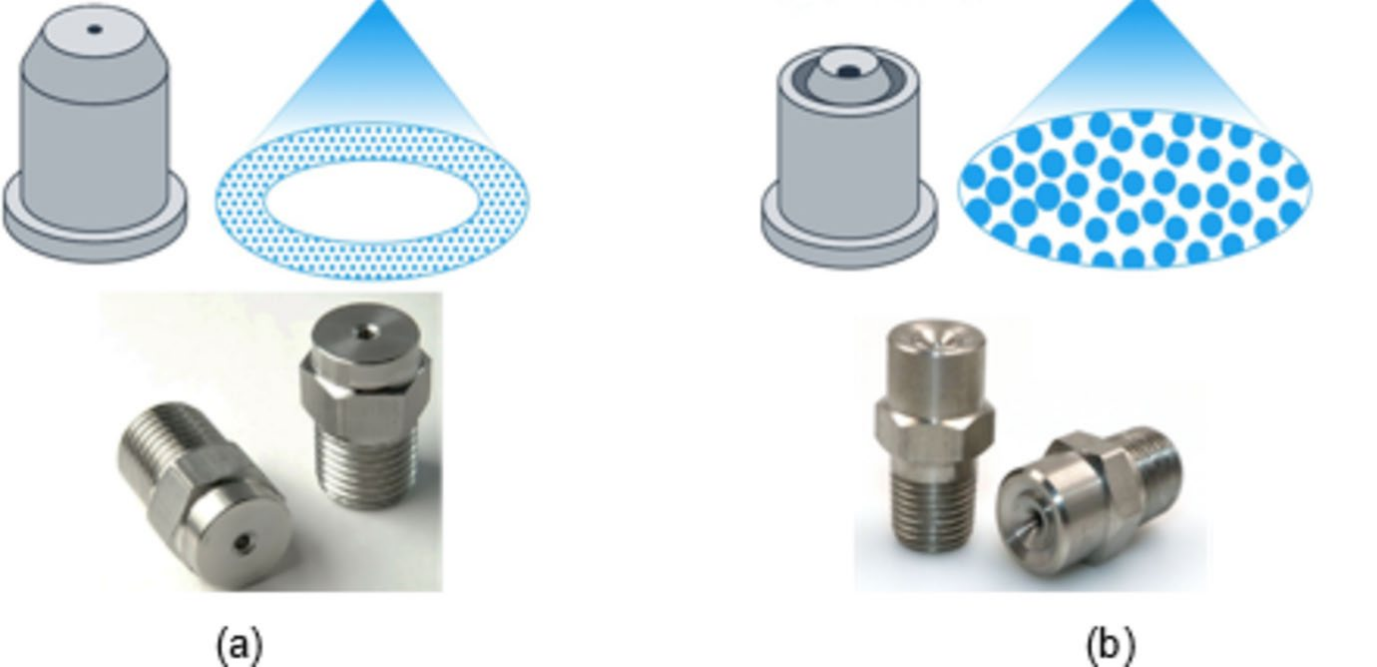
Nitrogen spraying nozzles. **a** Hollow-cone. **b** Full-cone.

The freezing experiment was designed using Minitab software utilizing Taguchi L_8_ (4^1, 2^2) array (Table 2). Litchi fruits were frozen under temperatures of − 40, − 50, − 60, and − 70 °C using the liquid nitrogen tunnel freezer at a Food Science College Laboratory at South China Agricultural University, Guangzhou, China. Two nitrogen spraying nozzle structures (Hollow-cone and full-cone) (Fig. 4) were experimented with, in addition to two distributing fan speeds of 800 and 1200 rpm.

The PLC unit allows for precise manual adjustment of the freezing temperature, which is continuously monitored by sensors installed throughout the tunnel. A control valve is integrated into the nozzle tubing line to regulate the spraying duration based on real-time temperature measurements. Additionally, five calibrated sensors are positioned along the freezing tunnel to monitor and display temperature variations during the experiment.

The hollow-cone nozzles generate a ring-shaped spray pattern, distributing the liquid in a hollow-cone formation, whereas the full-cone nozzles produce a uniform spray pattern that covers a circular area more evenly.

**Table 2:** Experimental design.

Parameter	Levels	Values
Temperature, °C	4	− 70, − 60, − 50, − 40
Fan speed, rpm	2	800, 1200
Nozzle type	2	Hollow-cone, full-cone

## Measurements

### Crack ratio

After the freezing experiment, the cracked fruits were collected, and the crack ratio was calculated as follows (Eq. 4):

4 $$\:\text{C}\text{r}\text{a}\text{c}\text{k}\:\text{r}\text{a}\text{t}\text{i}\text{o},\:{\%}=\frac{{\text{N}}_{crack}}{{\text{N}}_{total}},$$

where; $\:{\text{N}}_{crack}$ is the number of cracked fruits and $\:{\text{N}}_{total}$ is the total number of frozen fruits.

### Freezing time

The total freezing time for every treatment was derived from the data collected by the data logger used to monitor the fruit’s core temperature.

### Nitrogen consumption

The amount of liquid nitrogen consumed was calculated using the meter connected to the nitrogen pipe between the freezer and the tank.

### Litchi core temperature change

The core temperature change during the experiment was measured using the temperature sensors attached to a data logger as shown above (Fig. 3).

## Results and discussion

### Taguchi analysis

According to the Taguchi technique, the experimental factors were optimized using Minitab 18 software by calculating the S/N ratios (smaller is a better function) as the experiment seeks to lower crack ratio, less time, and lower nitrogen consumption. A residual plot was used to check the significance of the parameters in the measured values.

### S/N ratio and residual plot for crack ratio

**Fig. 5:**
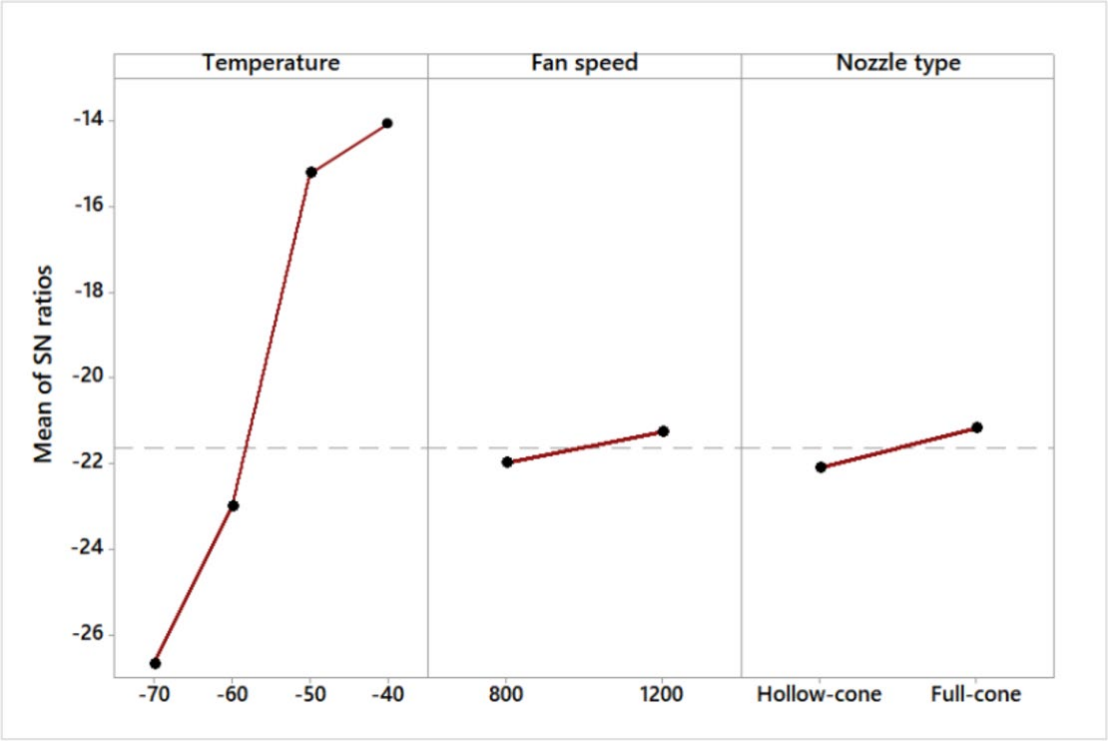
S/N ratio effect plot for crack (smaller is better).

**Fig. 6:**
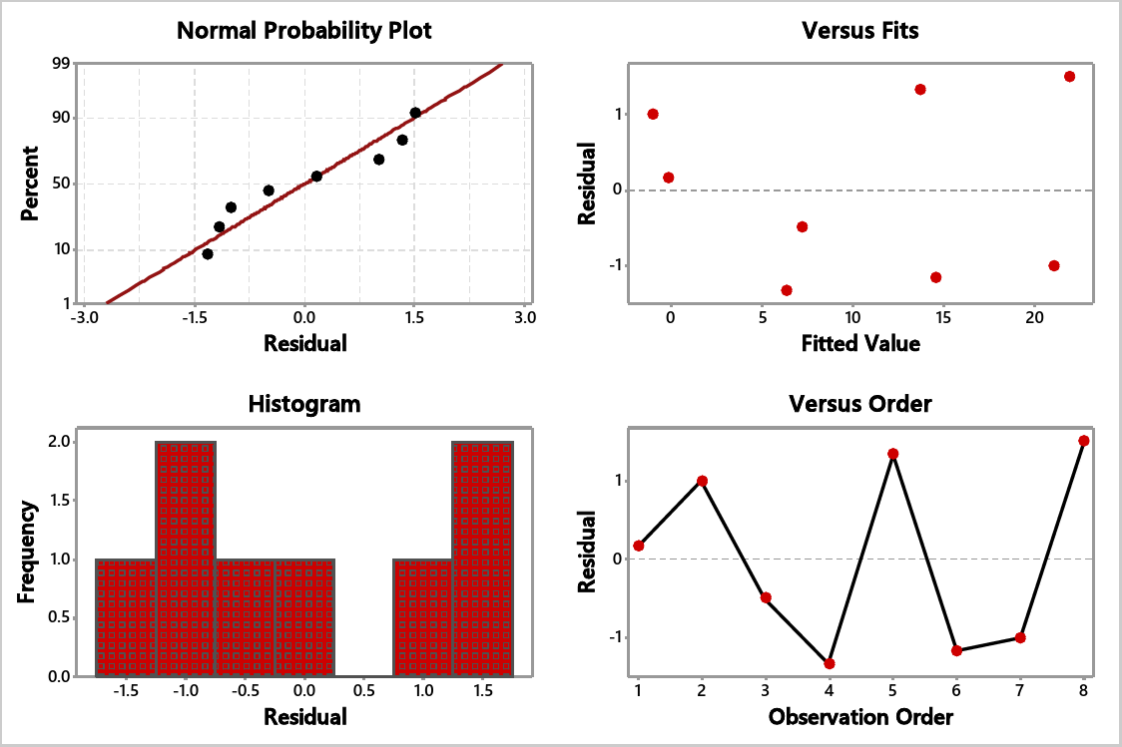
Residual plot for crack.

Table 3 shows the S/N ratios, illustrating that the freezing temperature is the most influential factor for a lower crack ratio, followed by nozzle type and fan speed. Also, Fig. 5 for S/N ratios Shows that the best combination for lower crack is −40 ºC, 1200 rpm, and full cone nozzle type. From the normal probability plot (Fig. 6), it is obvious that the residuals fall close to the straight line, reflecting the significance of the experimented variables.

**Table 3:** Crack S/N ratios (smaller is better).

Level	Temperature S/*N*	Fan speed S/*N*	Nozzle type S/*N*
1	− 26.69	− 22.01	− 22.11
2	− 23.01	− 21.28	− 21.17
3	− 15.23		
4	− 13.98		
Rank	1	3	2

### S/N ratio and residual plot for freezing time

**Fig. 7:**
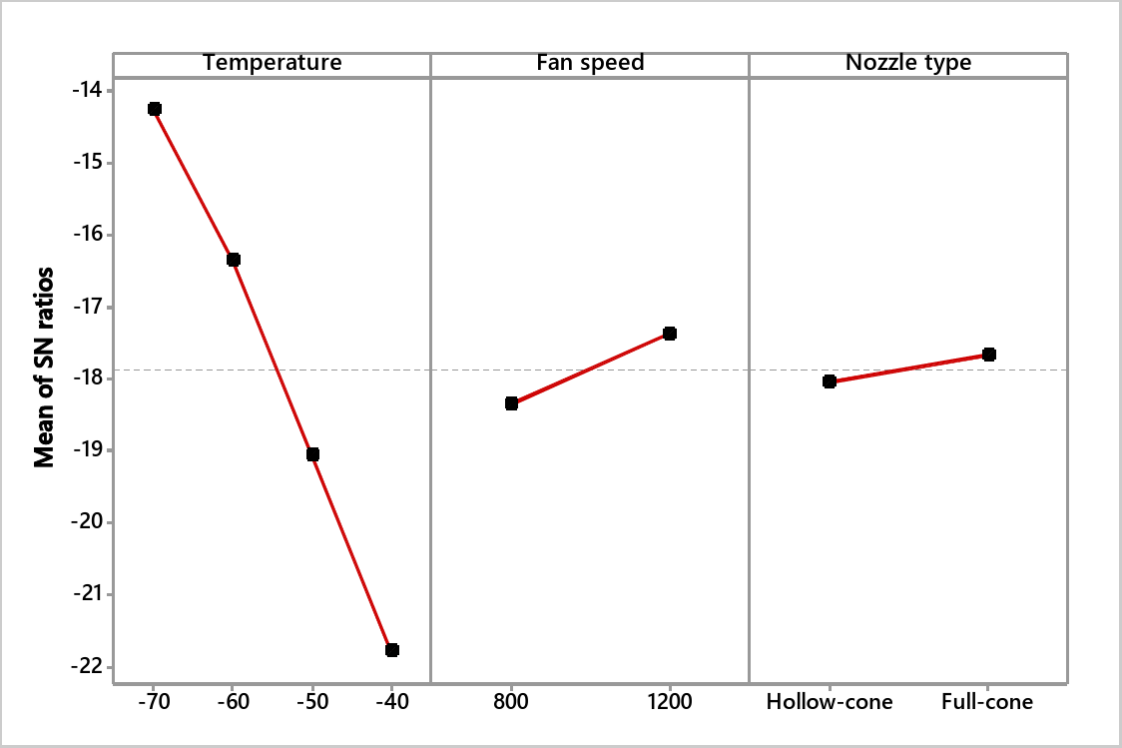
S/N ratio effect plot for time (smaller is better).

**Fig. 8:**
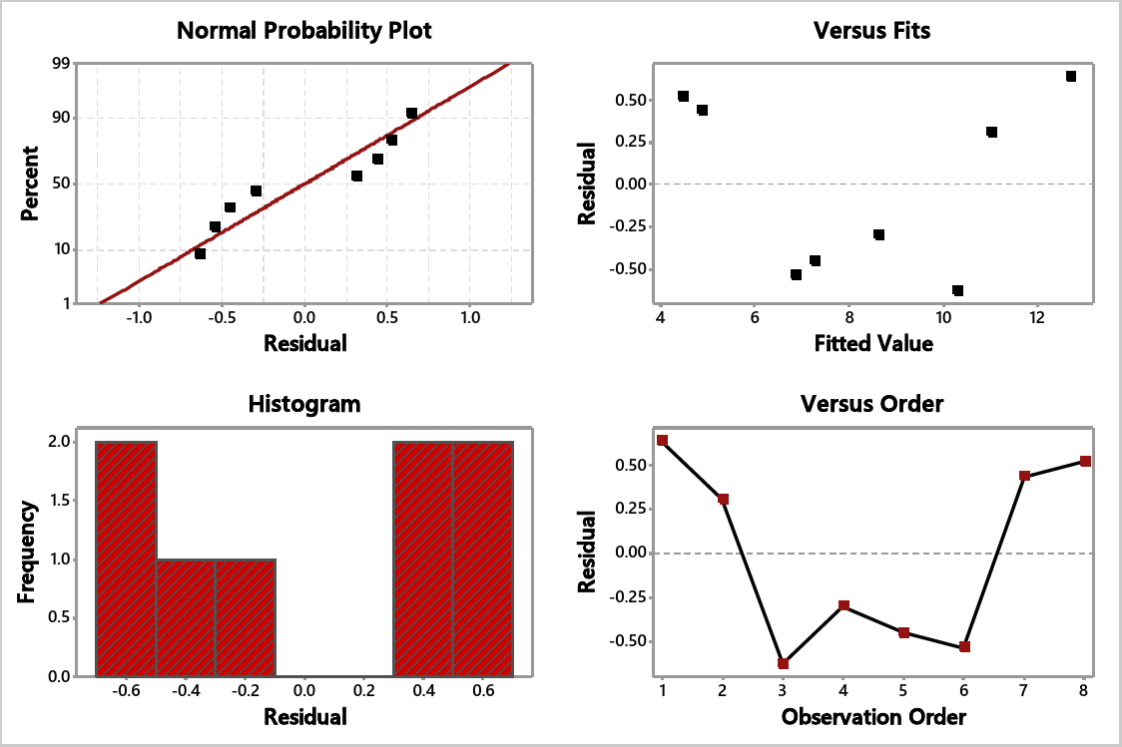
Residual plots for time.

As the experiment seeks to reduce the freezing time, the S/N smaller is better function was used for time analysis. As shown in Table 4, the sequence of the effective parameters for optimal freezing time was as follows: freezing temperature, fan speed, and, at last, the nozzle type. Figure 7 illustrates that the full cone nozzle can achieve the lowest freezing time under − 70 ºC and 1200 rpm. The residual plots are shown in Fig. 8, which reflects the significance of the experimented variables as the normal probability plot residuals are allocated close to the straight line.

**Table 4:** Time S/N ratios (smaller is better).

Level	Temperature S/*N*	Fan speed S/*N*	Nozzle type S/*N*
1	− 14.26	− 18.36	− 18.05
2	− 16.36	− 17.38	− 17.68
3	− 19.06		
4	− 21.79		
Rank	1	2	3

### S/N ratio and residual plot for nitrogen consumption

**Fig. 9:**
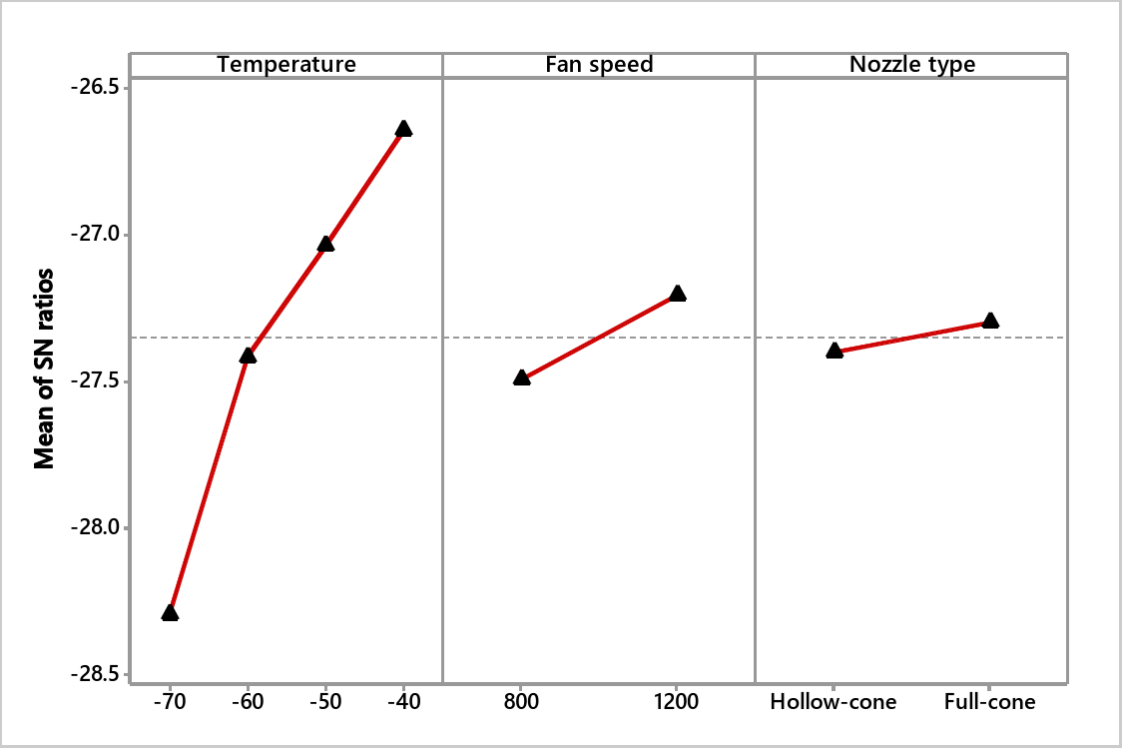
S/N ratio effect plot for nitrogen.

The lower is better function was used, as shown in Table 5, which reveals that the order of the effective factors is freezing temperature followed by the fan speed and, lastly, the nozzle type. The lowest nitrogen consumption was recorded using − 40 ºC with 1200 rpm fan speed and the full cone nozzle, as illustrated by Fig. 9.

**Fig. 10:**
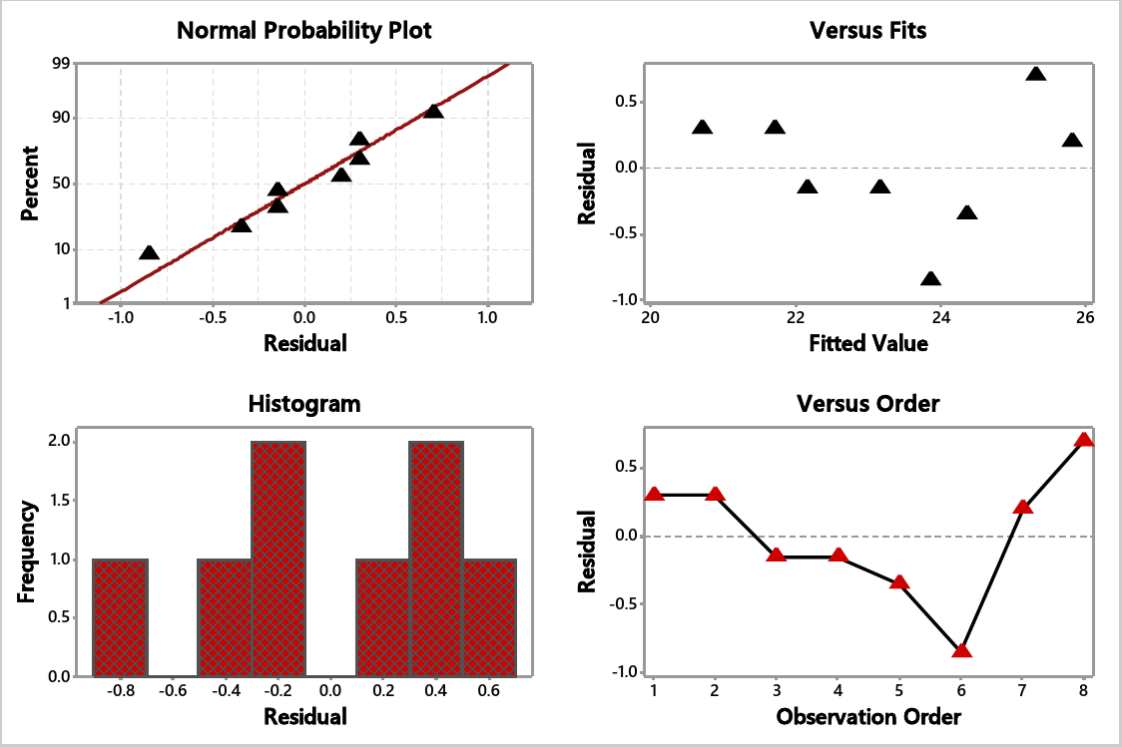
Residual plots for nitrogen.

Figure 10 introduces the residual plots for nitrogen consumption. The residuals are close to the straight line, mirroring the variables’ significance.

**Table 5:** Nitrogen S/N ratios (smaller is better).

Level	Temperature S/*N*	Fan speed S/*N*	Nozzle type S/*N*
1	− 28.30	− 27.68	− 27.59
2	− 27.42	− 27.21	− 27.30
3	− 27.40		
4	− 26.65		
Rank	1	2	3

### Results prediction and confirmation tests

Confirmation tests were performed in order to validate Taguchi’s optimal predicted parameters. The optimum results were predicted using Taguchi analysis, and it was almost the same as the experimental results (see Table 6).

In order to guarantee the reliability and accuracy of the Taguchi method for analyzing and predicting the results, some random experiments were established, and their results were compared to the predicted ones. The results are introduced in Table 7, which reveals a great agreement between the experimental and predicted results. Similar findings were recorded by^36–39^.

**Table 6:** Confirmation of the optimum parameters.

Measured value	Crack, %	Time, min	N2, L
Experimental result	0	11.80	21.00
Predicted result	0.42	11.85	21.00
Difference	0.42	0.05	0

**Table 7:** Predicted results validation.

Temperature	Fan speed	Nozzle type	Experimental results			Predicted results			Error %		
			Crack, %	Time, min	N2, L	Crack, %	Time, min	N2, L	Crack	Time	N2
− 40	1200	Full-cone	0	11.80	21	0.42	11.85	21	0	0.42	0
− 50	1200	Full-cone	5	8.33	22	5.42	8.28	22	8.35	0.57	0
− 60	1200	Full-cone	13.33	5.85	23	13.75	5.86	23	3.13	0.21	0
− 70	800	Hollow-cone	23.33	5.67	26	22.08	5.88	26.5	5.35	3.75	1.92

### Analysis of variance (ANOVA)

The analysis of variance was accomplished at 95% confidence level and 5% significance level to determine the significance and the influence rate of each parameter on the experimental results. Regression analysis was performed to introduce the relationship between the experimental parameters and the measured values. The ability of developed models is checked through the coefficient of determination (R^2^)^40^. When the coefficients are close to one, this reflects a good fit between the independent and dependent experimental variables.

### ANOVA for crack

Table 8 shows that temperature is the most effective and significant factor in the crack ratio, contributing to 96.65%. These findings highlighted that temperature is the most important factor in freezing when seeking a lower crack.

The regression equations are illustrated in Eqs. (5, 6). Regression analysis showed a high value for R^2^ of 98.28%.

5 $$\text{Hollow-cone} = -29.49 - 0.7333 \quad \text{Temperature} - 0.00001\, \text{Fan speed},$$

6 $$\text{Full-cone} = -30.32 - 0.7333 \quad \text{Temperature} - 0.00001\, \text{ Fan speed}.$$

**Table 8:** ANOVA for crack.

Source	DF	Seq SS	Contribution (%)	Adj SS	Adj MS	F-Value	*P*-Value
Regression	3	539.042	98.28	539.042	179.681	76.14	0.001
Temperature	1	537.656	98.03	537.656	537.656	227.85	0.000
Fan speed	1	0.000	0.00	0.000	0.000	0.00	0.998
Nozzle type	1	1.386	0.25	1.386	1.386	0.59	0.486
Error	4	9.439	1.72	9.439	2.360		
Total	7	548.481	100.00				

### ANOVA for time

Table 9 shows that the freezing temperature achieved the highest contribution rate of 93%, followed by the distribution fan speed and the nozzle type.

The regression equations are illustrated in Eqs. (7, 8). It had a higher R^2^ of 96.43%.

7 $$\text{Hollow-cone} = 24.44 + 0.2462 \quad \text{Temperature} - 0.00231\,\text{ Fan speed},$$

8 $$\text{Full-cone} = 23.93 + 0.2462 \quad \text{Temperature} - 0.00231\, \text{Fan speed}.$$

**Table 9:** ANOVA for time

Source	DF	Seq SS	Contribution (%)	Adj SS	Adj MS	F-Value	*P*-Value
Regression	3	62.8459	96.43	62.8459	20.9486	35.97	0.002
Temperature	1	60.6144	93.00	60.6144	60.6144	104.08	0.001
Fan speed	1	1.7112	2.63	1.7112	1.7112	2.94	0.162
Nozzle type	1	0.5202	0.80	0.5202	0.5202	0.89	0.398
Error	4	2.3295	3.57	2.3295	0.5824		
Total	7	65.1754	100.00				

### ANOVA for nitrogen

Table 10 shows each parameter’s contribution rate, highlighting that temperature is the most important factor for lower nitrogen consumption, followed by fan speed and nozzle type.

The regression equations are illustrated in Eqs. (9, 10) and the value of R^2^ was 93.30%.

9 $$\text{Hollow-cone} = 17.40 - 0.1450 \quad \text{ Temperature}- 0.00187\,\text{Fan speed},$$

10 $$\text{Full-cone} = 17.15 - 0.1450 \quad \text{Temperature}- 0.00187 \,\text{Fan speed}.$$

**Table 10:** ANOVA for nitrogen

Source	DF	Seq SS	Contribution (%)	Adj SS	Adj MS	F-Value	*P*-Value
Regression	3	22.2750	93.30	22.2750	7.4250	18.56	0.008
Temperature	1	21.0250	88.06	21.0250	21.0250	52.56	0.002
Fan speed	1	1.1250	4.71	1.1250	1.1250	2.81	0.169
Nozzle type	1	0.1250	0.52	0.1250	0.1250	0.31	0.606
Error	4	1.6000	6.70	1.6000	0.4000		
Total	7	23.8750	100.00				

### Effect of experimental parameters on the measured values

#### Effect of experimental parameters on the crack ratio

**Fig. 11:**
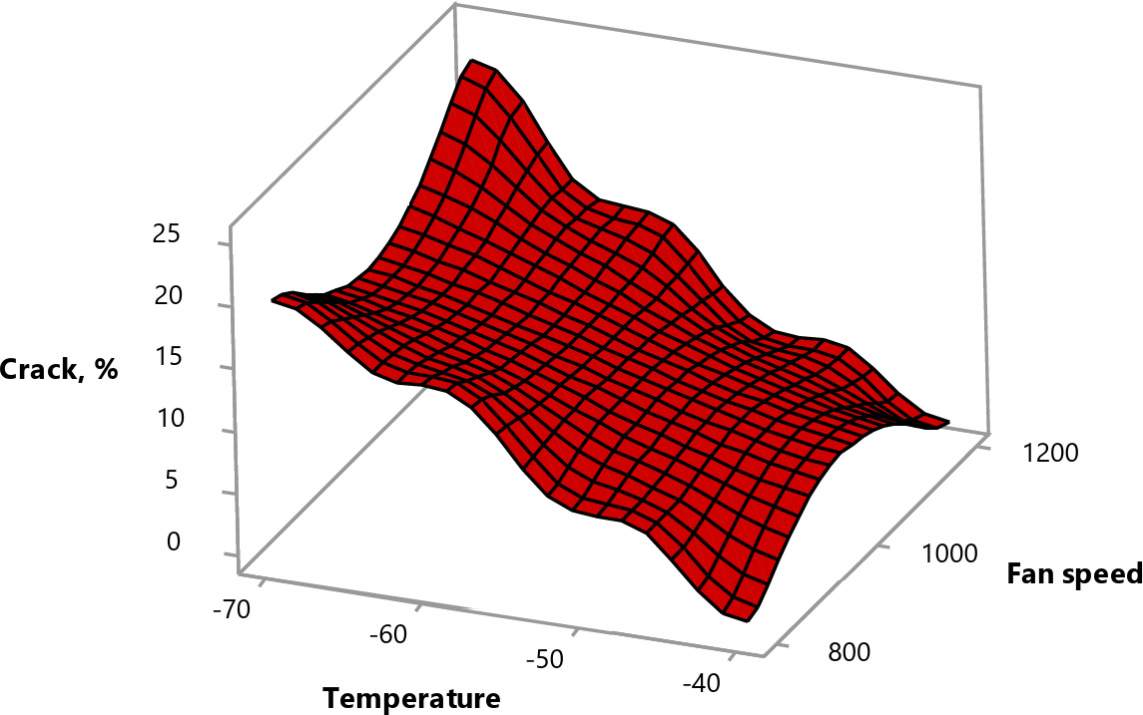
Surface plot of crack vs. fan speed, temperature.

No cracking was observed at − 40 °C, as the freezing rate and thermal stress on the fruit tissues were likely balanced facilitating more uniform heat transfer. This reduced mechanical stress on the fruit cell walls and prevented cracks. However, the crack ratio increased progressively as the temperature decreased from − 50 to −70 °C and the fan speed reduced from 1200 to 800 rpm (Fig. 11). This might be due to the higher expansion in volume of the fruit pulp than the peel due to the fast freezing, as the water content of the pulp is higher than the peel. The faster freezing rate leads to more irregular ice crystal formation. These crystals disrupt cell walls and create internal stresses, increasing the crack.

Additionally, the full-cone nozzles resulted in fewer cracks compared to the hollow-cone nozzles, as they provide a more uniform distribution of nitrogen promoting smaller, more evenly distributed ice crystals, which are less likely to puncture cell walls.

#### Effect of experimental parameters on freezing time

**Fig. 12:**
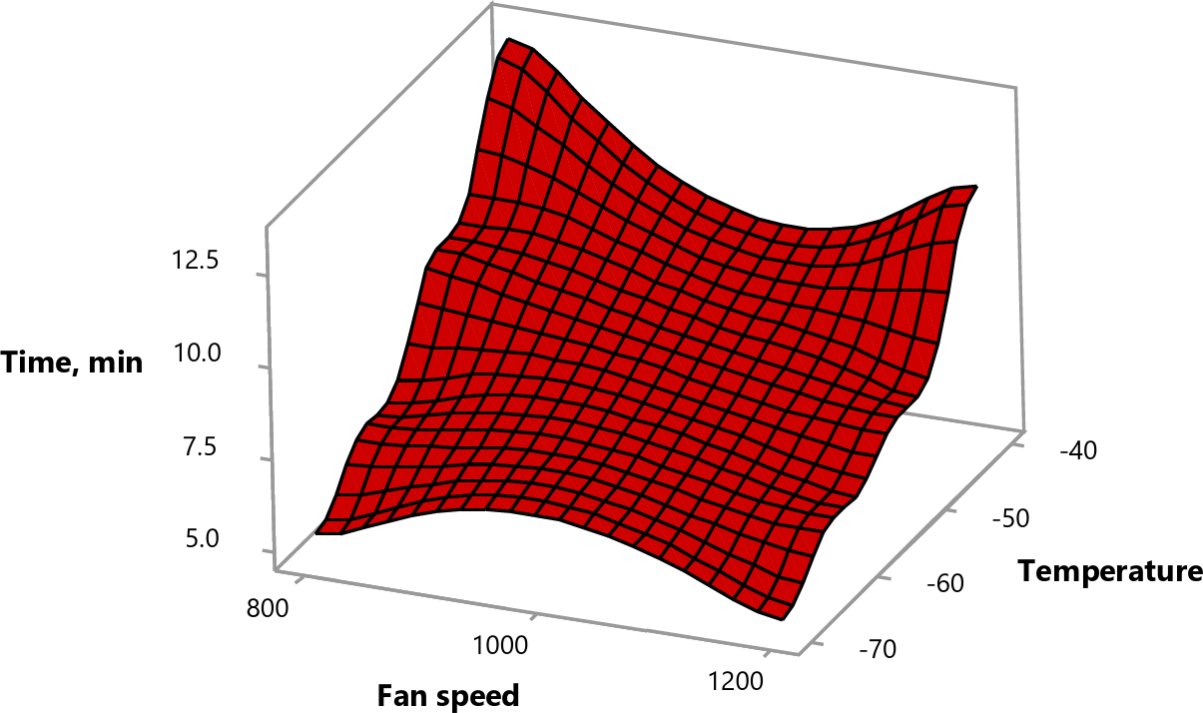
Surface plot of time vs. fan speed, temperature.

The freezing time decreased as the temperature decreased and the fan speed increased (see Fig. 12) due to the increased freezing rate, which accelerated the reduction in fruit temperature. Additionally, increasing the fan speed enhances the convective heat transfer inside the freezing tunnel, leading to more even and efficient cold air distribution around the fruit. This accelerates heat removal from the fruits, further dropping freezing time.

Utilizing full-cone nozzles reduced the freezing time as they generate a more uniform and wider spray pattern, covering a larger surface area of the fruits, enabling more consistent and efficient heat removal.

#### Effect of experimental parameters on nitrogen consumption

**Fig. 13:**
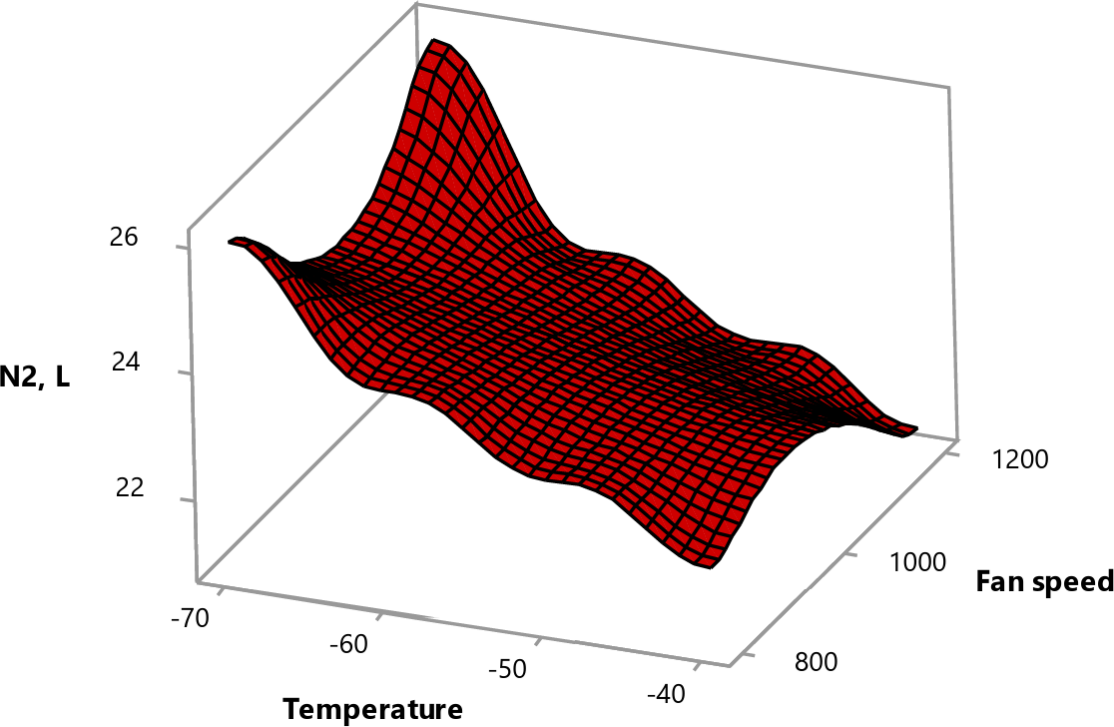
Surface plot of nitrogen consumption vs. fan speed, temperature.

It is noticed from Fig. 13 that nitrogen consumption increased while the freezing temperature decreased. This can be attributed to the longer spraying duration required to achieve and maintain lower temperatures within the freezing tunnel. Besides, the liquid nitrogen evaporates faster at lower temperatures.

Meanwhile, using the full-cone nozzles and increasing the fan speed from 800 to 1200 decreased the consumption. This could be attributed to the higher fan speed and full-cone nozzles uniform and wide spraying effectively distributes the nitrogen to the fruits while moving inside the tunnel and decreases nitrogen loss inside the ambient tunnel space which leads to a reduction in the nitrogen consumption.

#### Effect of the experimental parameters on core temperature change

**Fig. 14:**
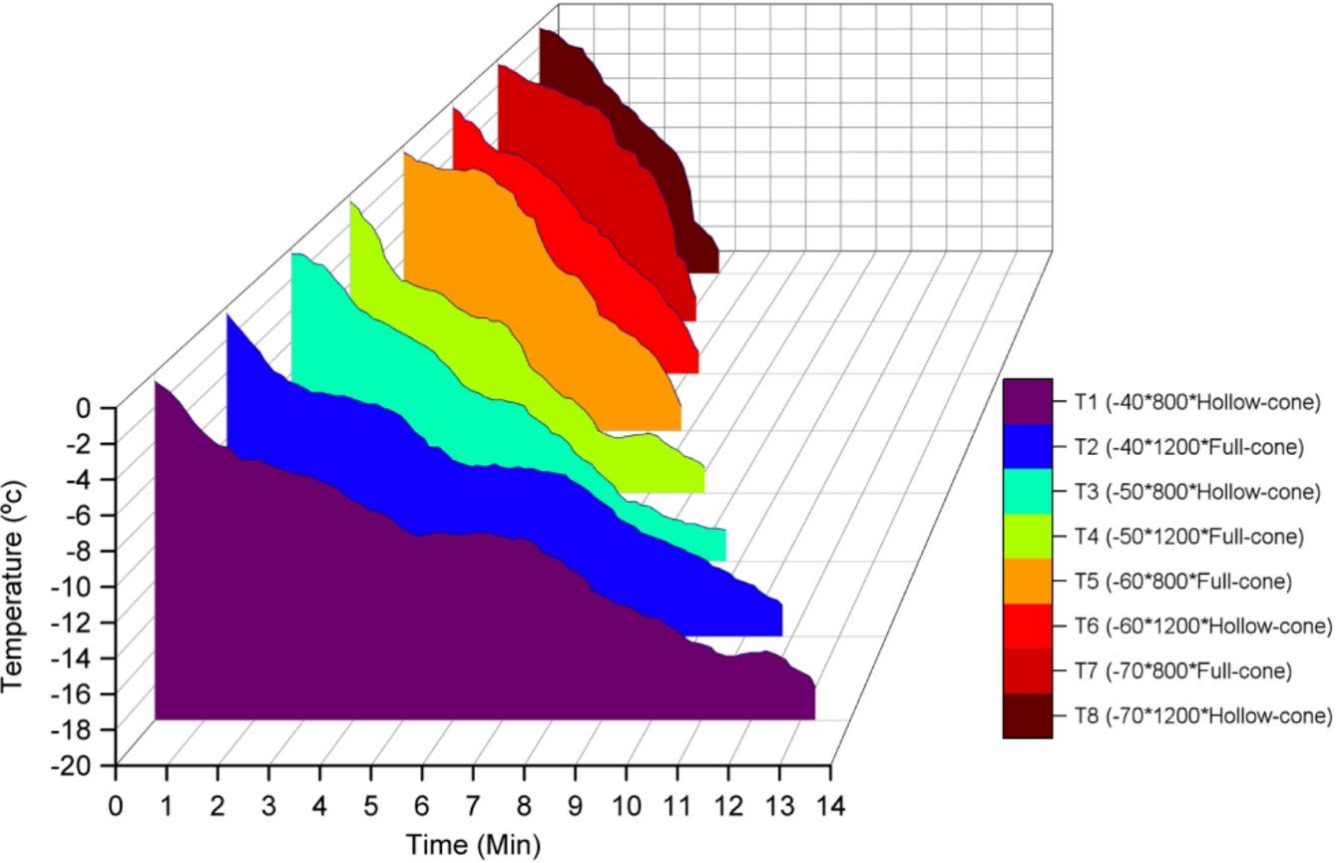
Litchi core temperature change.

Figure 14 illustrates the change in Litchi core temperature during the freezing experiments, which was recorded by the core temperature monitoring system. It can be noticed that the core temperature decreased at different rates for all the experimental parameters. It can also be seen that the freezing time decreased while the freezing temperature decreased and the fan speed increased. Besides, using the full-cone nozzles decreased the freezing time compared to the hollow-cone nozzles.

## Conclusion

This study utilized the Taguchi technique to evaluate the impact of various liquid nitrogen quick-freezing parameters on litchi fruit cracking, nitrogen consumption, and freezing time. Experiments were conducted under different freezing temperatures (− 40, − 50, − 60, − 70 °C), two types of nitrogen spray nozzles (Hollow-cone and Full-cone), and two distributing fan speeds (800 and 1200 rpm). Before freezing, litchis were soaked in a solution and precooled to protect the peel from cracking and maintain their quality. The experimental results demonstrated that higher temperatures and increased fan speeds significantly reduced the crack ratio, freezing time, and nitrogen consumption. Replacing hollow-cone nozzles with full-cone ones led to more than a 15% reduction in crack ratio, an 8% decrease in freezing time, and a 4% lower nitrogen consumption. This highlights the superior efficiency and performance of full-cone nozzles in the experimental results. The optimal freezing conditions were attained at −40 °C, with a fan speed of 1200 rpm, and using full-cone nozzles. These conditions resulted in zero crack and the lowest nitrogen consumption of 21 L. The Taguchi method represented good reliability for experimental design and analysis as the predicted results aligned closely with the experimental data. The study presented innovative pre-treatments and optimized freezing parameters to prevent fruit cracking during the freezing process, eliminating the need for packing, which reduced freezing time, costs, and nitrogen consumption. The authors may mention that further studies are needed to examine the impact of the freezing process on the quality of the fruits after thawing. Additionally, they express their intention to simulate heat transfer dynamics within the freezing tunnel and the fruits. Besides, Our following research will be devoted to examining the influence of broader selection of nozzle designs. This study serves as a novel and valuable reference for the freezing industry and research, offering significant contributions to nitrogen conservation and the reduction of fruit peel cracking.

## Data Availability

The datasets used and/or analyzed during the current study are available from the first and corresponding authors upon reasonable request.
